# Generation, characterization, and use of EKLF(Klf1)/CRE knock-in mice for cell-restricted analyses

**DOI:** 10.3389/frhem.2023.1292589

**Published:** 2024-01-19

**Authors:** Li Xue, Kaustav Mukherjee, Kevin A. Kelley, James J. Bieker

**Affiliations:** 1 Department of Cell, Developmental, and Regenerative Biology, Mount Sinai School of Medicine, New York, NY, United States; 2 Black Family Stem Cell Institute, Mount Sinai School of Medicine, New York, NY, United States; 3 Friedman Brain Institute, Mount Sinai School of Medicine, New York, NY, United States; 4 Tisch Cancer Institute, Mount Sinai School of Medicine, New York, NY, United States; 5 Mindich Child Health and Development Institute, Mount Sinai School of Medicine, New York, NY, United States

**Keywords:** CRE/LoxP, EKLF/KLF1, fetal liver, bone marrow, erythroid cells, macrophage cells

## Abstract

**Introduction:**

EKLF/Klf1 is a tissue-restricted transcription factor that plays a critical role in all aspects of erythropoiesis. Of particular note is its tissue-restricted pattern of expression, a property that could prove useful for expression control of a linked marker or enzymatic gene.

**Methods and results:**

With this in mind, we fused the CRE recombinase to the genomic EKLF coding region and established mouse lines. We find by FACS analyses that CRE expression driven by the EKLF transcription unit recapitulates erythroid-restricted expression with high penetrance in developing embryos. We then used this line to test its properties in the adult, where we found EKLF/CRE is an active and is a robust mimic of normal EKLF expression in the adult bone marrow. EKLF/CRE is also expressed in erythroblastic island macrophage in the fetal liver, and we demonstrate for the first time that, as seen during embryonic development, EKLF is also expressed in adult BM-derived erythroblastic island macrophage. Our data also support lineage studies showing EKLF expression at early stages of hematopoiesis.

**Discussion:**

The EKLF/CRE mouse lines are novel reagents whose availability will be of great utility for future experiments by investigators in the red cell field.

## Introduction

The ability to selectively ablate genes within specific tissues or cell types has provided a powerful genetic approach to enable molecular, biochemical, and cellular analyses of gene function (1). Foremost among these techniques is the use of the CRE recombinase, which is a phage-derived protein that targets recombination between two loxP sites (2). Given the availability of a “floxed” target gene, directed expression of CRE will efficiently remove the intervening DNA sequence at the cell(s) of interest (3).

Erythroid Krüppel-like Factor (EKLF; KLF1) is a zinc finger transcription factor that plays a critical role in all aspects of erythropoiesis (4–11). EKLF’s expression pattern makes it appealing for use as a driver for restricted CRE recombinase expression, as it is highly restricted throughout early development and in the adult. EKLF mRNA first appears at the neural plate stage (E7.5), localized to the earliest morphologically identified erythroid cells in the blood islands of the yolk sac (12). Expression switches to the fetal liver by E9.5, then ultimately to the adult bone marrow and splenic red pulp (12, 13). During hematopoiesis its levels are highest in the common myeloid and megakaryocyte/erythroid progenitors, after which it remains elevated only in the erythroid cell (14, 15). Additional studies demonstrate EKLF is also expressed in the unique central macrophage cell of the erythroblastic island (16–20).

As a result we have knocked-in the CRE recombinase into the EKLF transcription unit and here present its use for expression analyses in the developing embryo and in the adult.

## Methods

### EKLF-CRE mice

**FIGURE 1:**
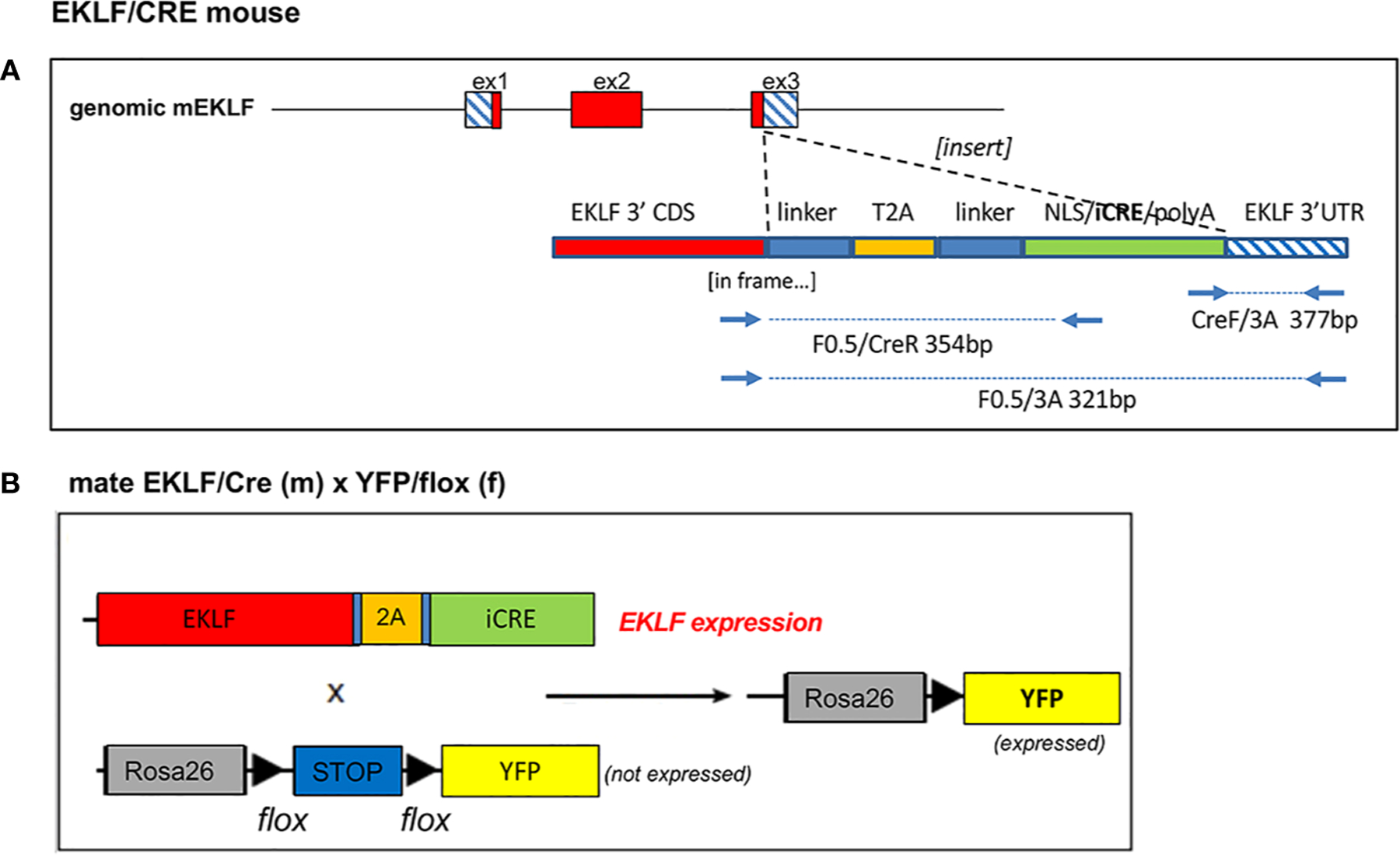
Design and use of EKLF/CRE genomic knock-in. (**A**) A Crispr/donor DNA approach was used to insert a linker/T2A/linker/CRE construct in frame into the EKLF coding region, as indicated. Also shown are diagnostic primers for genomic analysis; note the size for F0.5/3A is for WT, no insert. Sequences for donor DNA, Crispr RNA, and primers are in Supplementary Table S1. (**B**) Schematic of the mouse cross between EKLF/CRE and Rosa26/stop/YFP strains used in the present studies.

Donor DNA (IDT) and gRNA (IDT; designed via crispr.mit.edu) (as a Crispr Cas9 RNP) (21, 22) were co-microinjected into 1- to 2-cell mouse embryos, implanted into pseudo-pregnant females, and allowed to come to term (sequences are shown in Supplementary Table S1). Out of 84 pups, 12 were positive by genomic PCR genotyping (using 5’ and 3’ primer pairs as indicated in Figure 1, Supplementary Table S1), and 5 males were chosen to be further analyzed. One did not transmit via the germ line, and out of the remaining 4 lines we chose 2 for the extended analysis below. These were designated ‘EKLF-CRE’ mice.

To assess tissue-specificity of CRE activity, male EKLF-CRE mice were crossed with female R26R-YFP mice (kind gift from N Dubois (23)), all at 4–6 weeks of age.

Genotyping was performed on 20 ng genomic DNA isolated from tails by PCR analysis using the diagnostic oligonucleotide primers in Supplementary Table S1. These assessed the 5’ (F0.5/CreR) and 3’ (CreF/3A) junctions between the knock-in donor DNA and the EKLF gene (Figure 1, Supplementary Figure S1A). Use of the ‘outer’ primer pair (F0.5/3A) yields a small product in the absence of the knock-in (Figure 1, Supplementary Figure S1A). Subsequent experiments show that multiplex PCR will also work with these oligos (not shown), thus enabling single-lane monitoring of knock-in and WT alleles for hetero- and homo-zygosity. Expression levels of endogenous EKLF RNA do not decrease, but rather appear to be stabilized by the knock-in (Supplementary Figure S1B).

### Cellular analyses

Fetal livers were dissected from embryonic day E13.5 embryos or adult bone marrow and mechanically dispersed into single cells for fluorescence activated cell sorting (FACS). Briefly for bone marrow (BM), femurs were removed and the marrow was flushed by gently inserting a 23-gauge needle fitted with 3 ml syringe into the opening of the bone using 1 ml cold PBS/10%FBS. Fetal liver or bone marrow dissociated cells were filtered through a 70uM cell strainer (Falcon # 352350), and collected by centrifugation for 5 min at 1000rpm, and finally washed with PBS/10%FBS.

Cells to be analyzed for macrophage included the FWV peptide during the isolation procedure to help prevent aberrant macrophage/erythroid cell associations when used in combination with effective dispersal and singlet enrichment (17, 24).

Suspended cells were stained with the following antibodies: anti-mouse F4/80-PE (eBiosciences #12–4801-80), CD71-PE (eBioscience #12–0711-81), Ter119-APC (eBioscience #17–5921-81), CD44-FITC (Biorad #MCA89FT), anti-spectrin b1 (Santa Cruz #sc-374309) conjugated with AlexaFluor 647 (Invitrogen #Z11235), Sca1 (Ly6A/E)-PE (BD #BDB561076), B220 (CD45R)-PE (BD #BDB561878), CD3-PE, BD #BDB561799). Flow cytometry data was analyzed by FCS Express software, and gates were drawn based on unstained and single-color compensation controls from the same samples, using the same dyes and within the same experiment.

Erythroblastic island clusters were enriched from dispersed adult BM using a serum gradient (17).

For immunofluorescence of BM single cells or island clusters, cytospins were incubated with anti-spectrin b1 (Santa Cruz #sc-374309), anti-F4/80 (eBiosciences #12–4801-80), or anti-EKLF 7b2a (25) as described (17). Secondary antibodies included Alexa flour 568 goat anti-mouse IgG (Invitrogen #A11031) and Alexa flour 647 donkey anti-mouse IgG (Invitrogen #A-31571).

Photography was performed with a Zeiss Axio Imager.Z2(M) equipped with a Q-Imaging camera.

### Magnetic nanoparticle isolation of HSCs, T-cells, and B-cells

BM cells were obtained as above. Sca1+, CD3+, B220+ and Ter119+ BM cells were then isolated using the EasySep mouse PE positive selection kit (Cell Signaling Technologies #17656) according to the manufacturer’s instructions (detailed method available in (24)). 0.3ug of the selected PE-labeled antibodies (as used above for flow cytometry) were added for each isolation. For Ter119+ cells we used 0.3ug of anti-mouse TER-119-PE antibody (BD #553673).

### RNA isolation and RT-qPCR

RNA was isolated from selected BM cells using Trizol and purified RNA was treated with Turbo DNase (Invitrogen #AM1907). cDNA was synthesized using the SuperScript IV VILO kit (Invitrogen #11756050) and real time qPCR was performed using a Qiagen QuantiTect SYBR Green RT-PCR Kit. Primers are listed in Supplementary Table S1.

## Results

### Establishment of EKLF-CRE mice

A Easi-Crispr-cas9 approach (21, 22) was used to knock in the iCRE coding segment into the 3’ sequence of the endogenous mouse EKLF gene, fused in-frame to the final leucine amino acid (Figure 1A). This entailed the design of a directed Crispr gRNA along with a single-stranded donor DNA. The donor DNA included a self-cleaving T2A sequence (26) flanked by linkers, adjacent to the NLS/iCRE sequence (derived from pCAG-iCRE (27)). The ultimate design (based on (28)) relies on the endogenous EKLF polyA signal. Two lines were more extensively analyzed.

### EKLF-CRE is expressed and functional in the erythroid lineage

**FIGURE 2:**
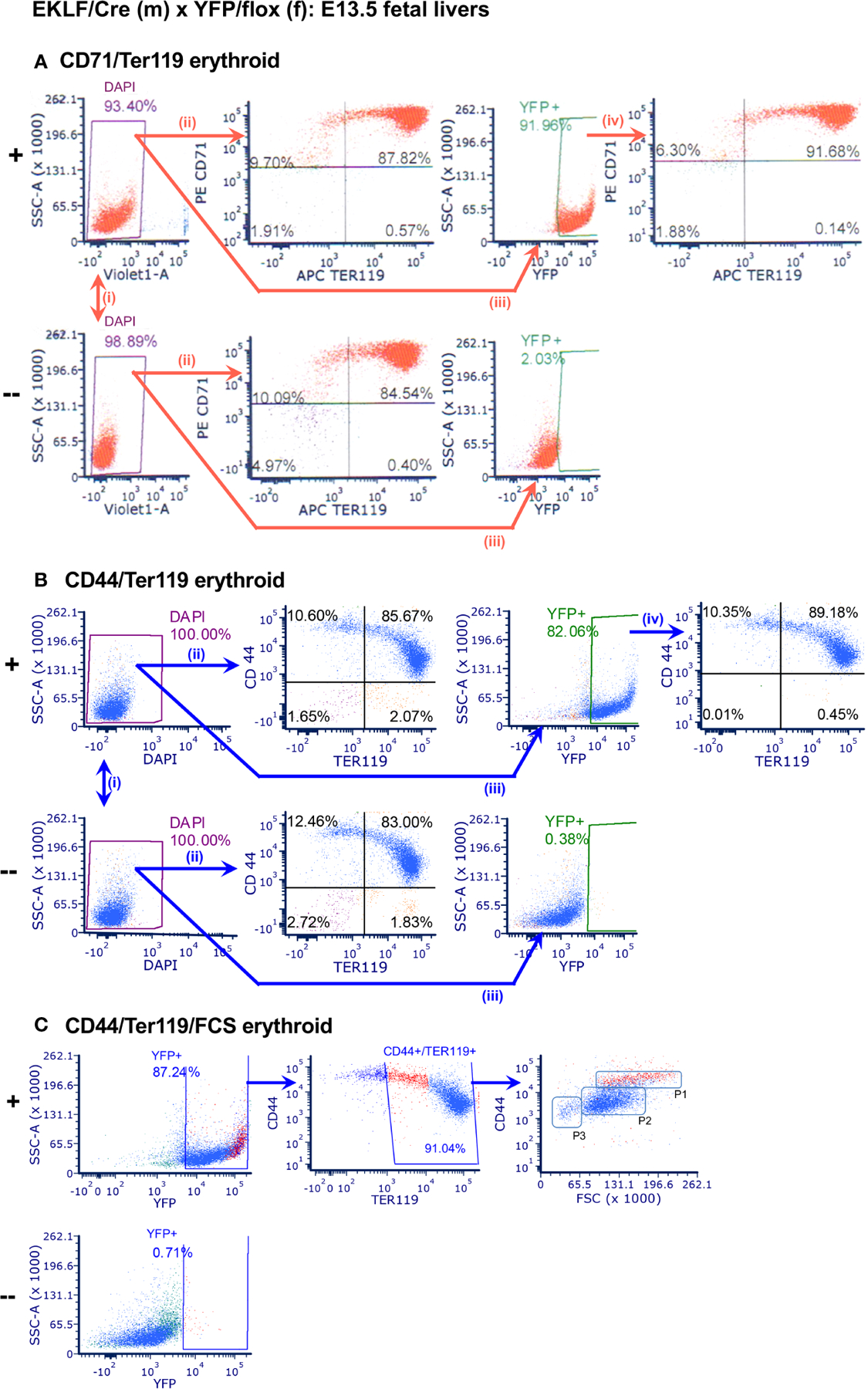
Analysis of CRE activity in E13.5 fetal liver erythroid cells. In each case, comparison is between YFP+ (+; *top*) or YFP− (−; *bottom*) littermates; n=9. (**A**) FACS analysis of YFP+ overlap with CD71/Ter119: (i) *live cells*, (+) 92.5 ± 1.8%, (−) 94.2 ± 5.2%; (ii) *CD71+/Ter119+ erythroid cells*, (+) 86.2 ± 1.8%, (−) 85.4 ± 3.6%; (iii) *YFP signal*, (+) 89.9 ± 2.3%, (−) 2.3 ± 0.9%; (iv) *overlap*, 90.4 ± 1.3%. (**B**) FACS analysis of YFP+ overlap with CD44/Ter119: (i) *live cells*, (+) 97.5 ± 4.2%, (−) 94.2 ± 9.1%; (ii) *CD44+/Ter119+ erythroid cells*, (+) 84.7 ± 1.3%, (−) 84.4 ± 2.8%; (iii) *YFP signal*, (+) 80.5 ± 1.8%, (−) 0.4 ± 0.3%; (iv) *overlap*, 88.6 ± 0.6%. (**C**) FACS analysis of YFP+ overlap with CD44/Ter119/FSC: *YFP signal*, (+) 85.9 ± 1.8%, (−) 0.7 ± 0.4%; *CD44+/Ter119+/FSC*, (+) 91.0 ± 0.2%. P1, P2, P3 regions indicate erythroblast maturation from less to more mature (31); P1 subpopulation is colored in red.

To test for CRE functionality, we crossed the EKLF-CRE mice with the Rosa26/stop/EYFP reporter line (29), which expresses YFP only if CRE is active and removes the flox-embedded stop sequence (Figure 1B). This yielded mice at the expected Mendelian ratios; we used littermate embryos for comparison. E13.5 embryos were harvested, and individual fetal livers cells were dispersed and analyzed by FACS. At this stage ~90% of cells are erythroid (30). Erythroid cells were monitored by CD71/Ter119 cell surface markers (Figure 2A) as these provide a good initial survey of immature to mature red cells. After proper gating and enrichment of singlets we find that the number of live cells are equivalent when comparing YPF+ to YFP- littermates (Figure 2Ai), and that the percentage of erythroid cells within this population is equivalent (Figure 2Aii). There is an excellent signal with respect to YFP detection, which is near absent in cells without EKLF-CRE (Figure 2Aiii). Importantly, we find the YFP+ cells overlap the CD71/Ter119 expression pattern identically to that seen endogenously (Figure 2Aiv). These data indicate that CRE expression driven by the EKLF transcription unit recapitulates erythroid-restricted expression with high penetrance in developing embryos.

To monitor CRE activity earlier in erythroid differentiation, we analyzed YFP overlap with the CD44 marker (Figure 2B). The CD44 sialoglycoprotein is expressed highest in proerythroblasts but then decreases as maturation proceeds (32). As before, there is no effect on live cell numbers (Figure 2Bi) or the CD44/Ter119 pattern (Figure 2Bii), and the YFP+ signal remains unique to the EKLF-CRE expressing cells (Figure 2Biii). These analyses again show complete overlap of YFP+ with the CD44/Ter119 range of expression seen endogenously (e.g., double positive cells are similar in number; Figure 2Biv). Combining these markers along with cell scatter properties (FSC) provides an accurate assessment of maturity of erythroid cells (31). As seen in Figure 2C, the CD44+/FSC profile within the Ter119+ subset shows that CRE expression is apparent in stages from proerythroblast to orthochromatic erythroblast, distributed as seen in prior fetal liver erythroid analyses (i.e., P2>P1>P3 (25);), and highest in the least mature population (P1; colored in red). This is a direct overlap with the known EKLF RNA and protein expression patterns (33, 34).

**FIGURE 3:**
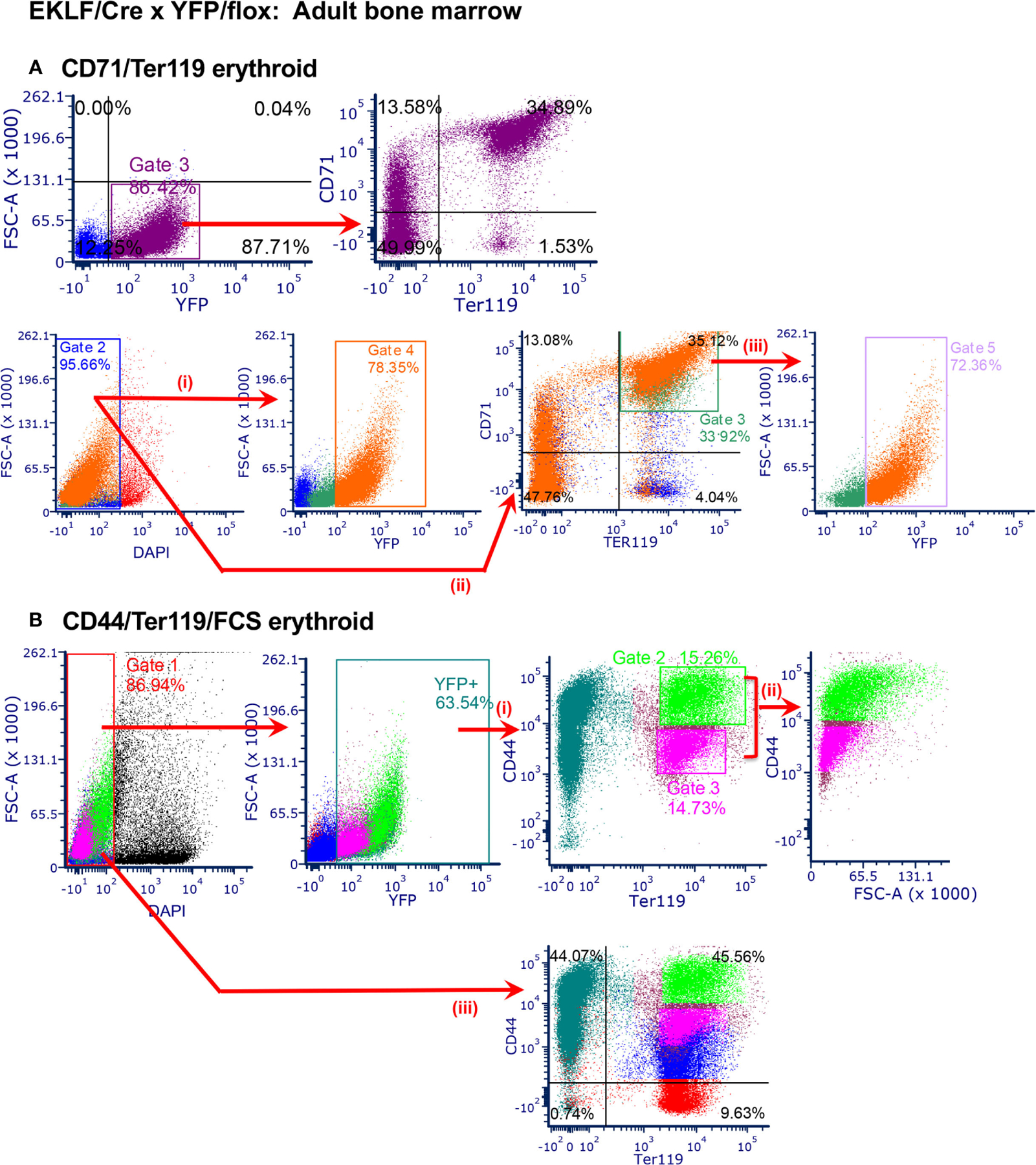
Analysis of CRE activity in adult bone marrow cells. n=2–5 analyses from 3 separate matings (**A**) *TOP*: FACS analysis of YFP+ overlap with CD71/Ter119, focusing on YFP+ cells: *YFP+*, 88.6 ± 2.2%; *YFP+/CD71+/Ter119+*, 32.8 ± 2.2%; *YFP+/CD71-/Ter119-*, 50.6 ± 0.6%. *BOTTOM*: FACS analysis of YFP+ overlap with CD71/Ter119, focusing on total cells, color coded throughout: (i) *live cells*, 94.8 ± 0.9%; *YFP+*, 81.0 ± 2.6%; (ii) *CD71+/Ter119+*, 35.1 ± 1.1%; *CD71-/Ter119-*, 43.5 ± 4.0%; (iii) *CD71+/Ter119+/YFP+*, 71.0 ± 1.5%. (**B**) FACS analysis of YFP+ (65.4 ± 1.9%) overlap with CD44/Ter119 (i), then selectively coupled to FSC (ii) (*top*) (*CD44hi*, 14.0 ± 1.2%; *CD44mid*, 14.2 ± 0.4%; *CD44lo*, 0.0%) (31). *Bottom* is same analysis after gating on total (YFP+ and −) cells (iii).

To assess whether EKLF-CRE is also functional in the adult, we performed the analogous study using bone marrow (BM) cells as our starting material. These data again show a direct overlap of YFP+ with CD71/Ter119 (Figure 3A *top*); however, in this case there are differences in detail compared to the fetal liver material. Although YFP+ overlaps with CD71+Ter119-, CD71+Ter119+, and CD71-Ter119+ cells as expected, there are a considerable number of YFP+ cells that are double negative, properties that had not been observed with fetal liver erythroid cells (likely these are F4/80+ cells, as explained below). To assess more subtle changes, we monitored subpopulations by gating on total live cells and color-coding the output (Figure 3A *bottom*, i and ii). These reproduce Figure 3A *top* (judged by the orange color YFP+ subset within the total cell population) but additionally show that not all CD71+Ter119+ cells are YFP+ (Figure 3A *bottom*, iii), observations likewise not seen with fetal liver-derived cells. Analysis of CD44/Ter119 expression shows that YFP+ cells segregate into discrete subpopulations encompassing different levels of CD44 and Ter119 (Figure 3Bi), and inclusion of FSC with selective gating (Ter119+ in combination with the CD44-lo to -hi (31)) shows that EKLF-CRE (YFP+) is most highly expressed in the least mature (ie, CD44+/Ter119+/FSC-mid to -hi) erythroid populations (Figure 3Bii), as perhaps more readily apparent by comparison to the total live cell population (i.e., both YFP+/−; Figure 3Biii).

Collectively these data demonstrate that EKLF-CRE is active and is a robust mimic of normal EKLF erythroid expression in the developing embryo and in the adult.

### EKLF-CRE is expressed and functional in erythroblastic island macrophage in the embryo and in the adult

**FIGURE 4:**
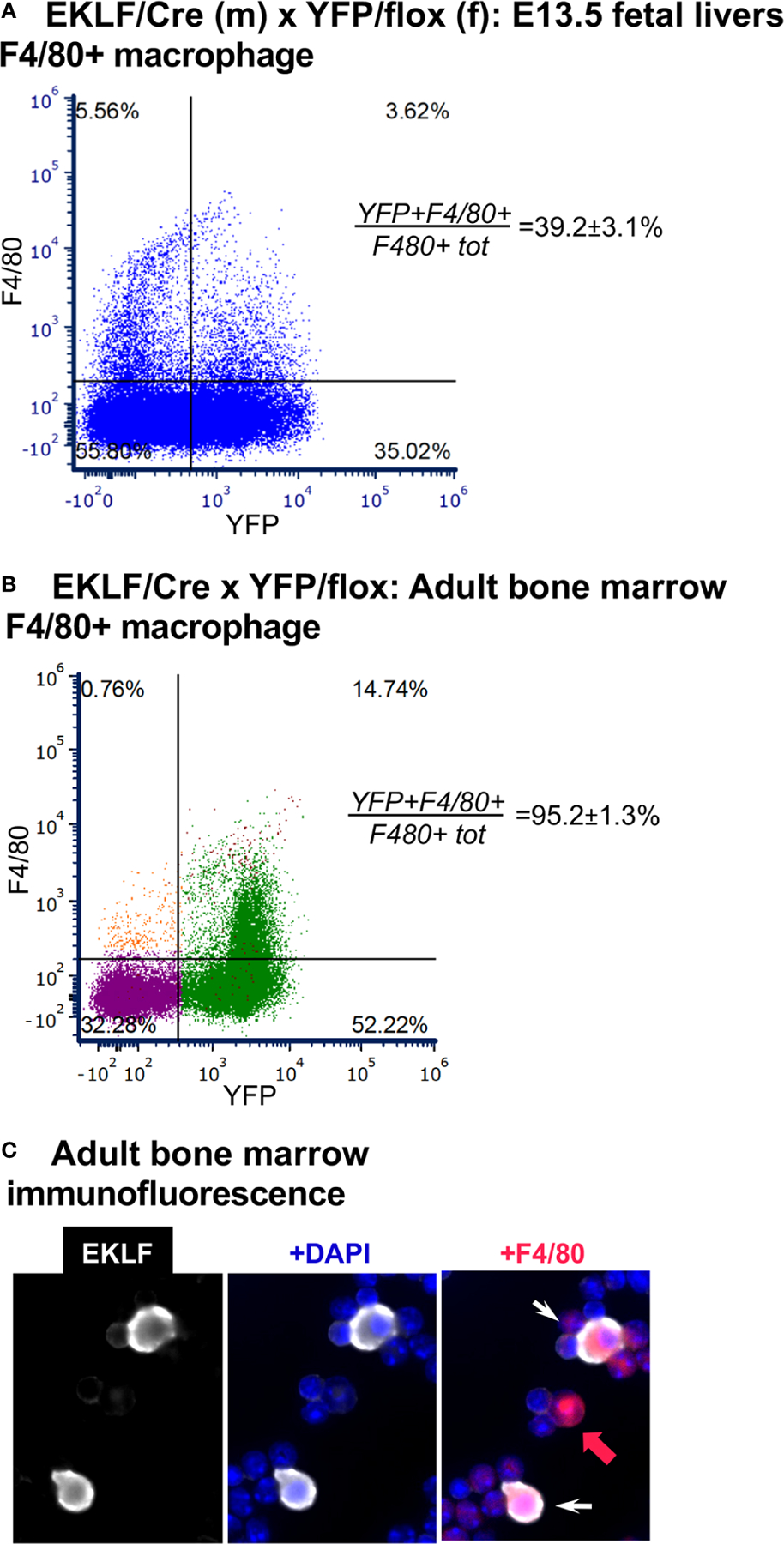
Analysis of CRE activity in F4/80+ macrophage. (**A**) FACS analysis of YFP+ overlap with F4/80 in E13.5 fetal liver cells, n=4. *F480+tot/live cells*=9.2 ± 0.9% (as previously observed (20)). (**B**) FACS analysis of YFP+ overlap with F4/80 in adult bone marrow cells, n=6. As BM has not been previously analyzed in the context of F4/80 and EKLF expression, forward/side scatter gates along with the DAPI- selection used for this experiment are shown in Supplementary Figure S2. *YFP+F480+/(F480+ tot)* negative control <2% (not shown); *F480+tot/live cells*=17.0 ± 1.1%. (**C**) Immunofluorescence analysis of EKLF protein (*white*) overlap with F4/80+ (*red*) in adult bone marrow cells (*white arrow*). As expected, not all cells are double positive (*red arrow*).

EKLF is also expressed within the central macrophage of the erythroblastic island (17, 18, 20, 35), a niche that provides a physiological support environment for erythroid maturation (36–39). F4/80 is an excellent marker for this cell (20). Using E13.5 fetal liver cells as our source we find that CRE is expressed in the F4/80+ cell population (Figure 4A); however, as seen in earlier studies, not all cells are positive, but rather 39 ± 3% are, which matches that previously seen by marked GFP+ (36% (17)). These results, using an independent approach from those used previously (16–20), provide additional support for EKLF’s expression in the F4/80+ central macrophage of the erythroblastic island.

One issue not hitherto addressed is whether EKLF is also expressed in the adult BM F4/80+ macrophage cell. As with our published fetal liver analyses (20), we included the FWV peptide to interfere with erythroid/macrophage interactions (17, 24) and used stringent forward- and side-scatter sorting prior to analysis (Supplementary Figure S2). Using YFP positivity as a surrogate marker for EKLF expression, we find that YFP expression is expressed in almost all (>90%) BM F4/80+ cells (Figure 4B). In this case it is also noteworthy that the total number of F4/80+ cells is ~17%, which is higher than that seen in the fetal liver, and serves to identify most of the large percentage of non-erythroid cells (i.e., double negative for CD71/Ter119) observed before (in Figure 3). To further verify that the YFP signal is a true mimic of endogenous EKLF expression, we performed immunofluorescent analysis of BM cells and find that indeed EKLF protein is colocalized in single F4/80+ cells (Figure 4C; cytoplasmic EKLF signal is expected (40)). These data show for the first time that, as seen during embryonic development, EKLF is also expressed in adult BM-derived erythroblastic island macrophage. The unexpected surprise, however, is that it is expressed in almost all F4/80+ cells.

**FIGURE 5:**
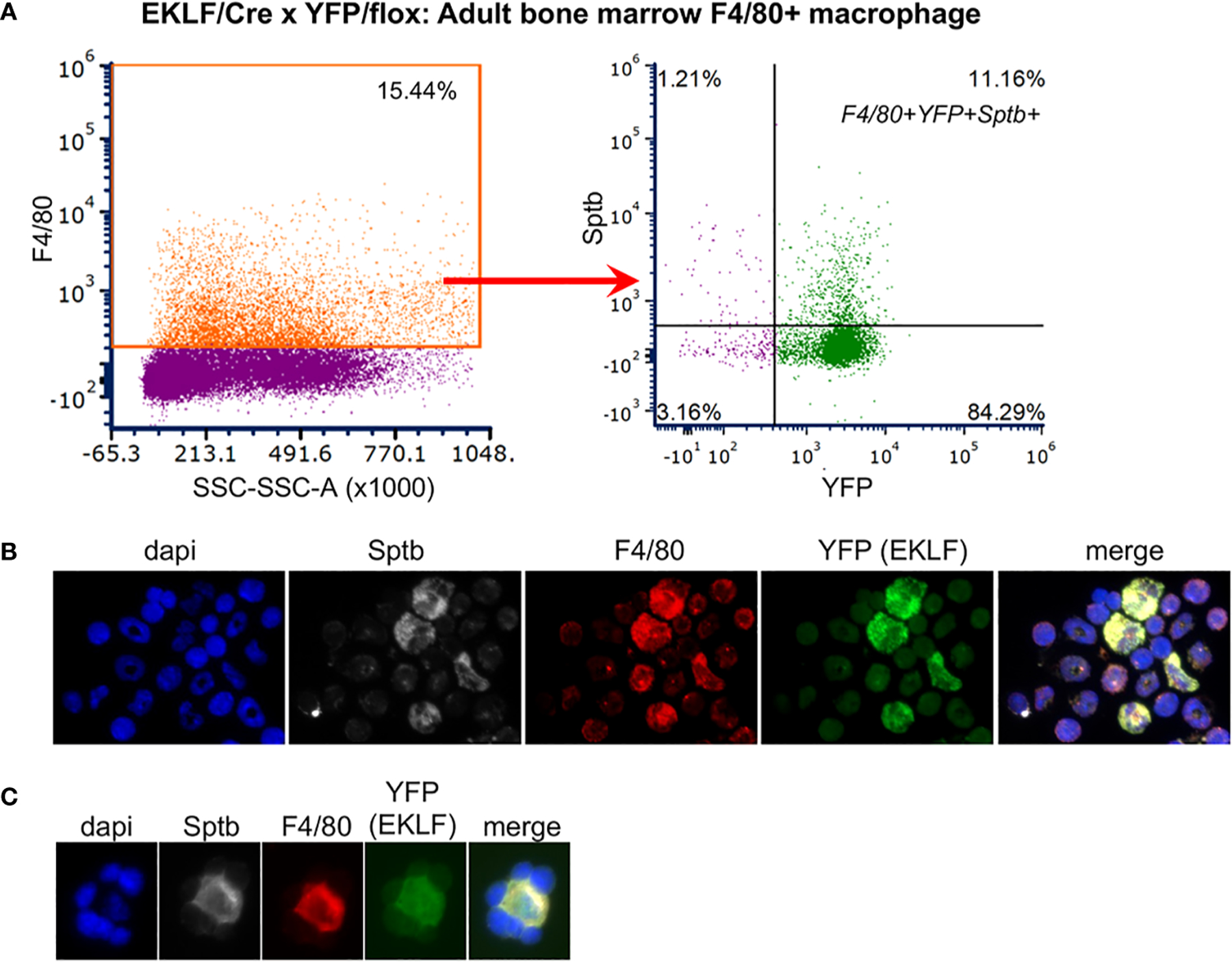
Co-expression of island macrophage markers in adult bone marrow cells. (**A**) FACS of F4/80+ cells gated for dual Sptb+ and YFP+ expression. Forward/side scatter gates along with the DAPI- selection used for this experiment are shown in Supplementary Figure S2. (**B**, **C**) Immunofluorescent analysis of overlapping expression, showing nuclear stain (dapi) in *blue*, F4/80 in *red*, YFP (EKLF) in *green*, Sptb in *white*. Visualization of single cells (**B**) or an erythroblastic island (**C**) are shown. Consistent with FACS analyses, not all cells are positive for all markers.

We had previously found that Adducin (Add2), ß-spectrin (Sptb), and to a lesser extent Adra2b, are cell surface markers enriched in EKLF-expressing fetal liver F4/80+ macrophage (20). To assess whether this is the case in adult BM, we performed FACS analyses for each of these. Although the levels of Add2 and Adra2b expression in F4/80+ BM cells were insignificant (not shown), we find that 11% of F4/80+ BM cells are positive for both Sptb+ and YFP+ (Figure 5A, Supplementary Figure S2). Immunofluorescent analyses show co-expression of EKLF/YFP, F4/80, and ß-spectrin in single cells (Figure 5B) and in erythroblastic islands (Figure 5C). These results demonstrate that expression of the Sptb surface marker in adult F4/80+ cells that express EKLF are as observed in the developing embryo.

Of relevance to the present resource, these data demonstrate that EKLF-CRE can be used to target a specific subset of macrophage, one that is highly enriched within the island niche, in both fetal liver and adult BM cells.

### Lineage tracing by EKLF-CRE

**FIGURE 6:**
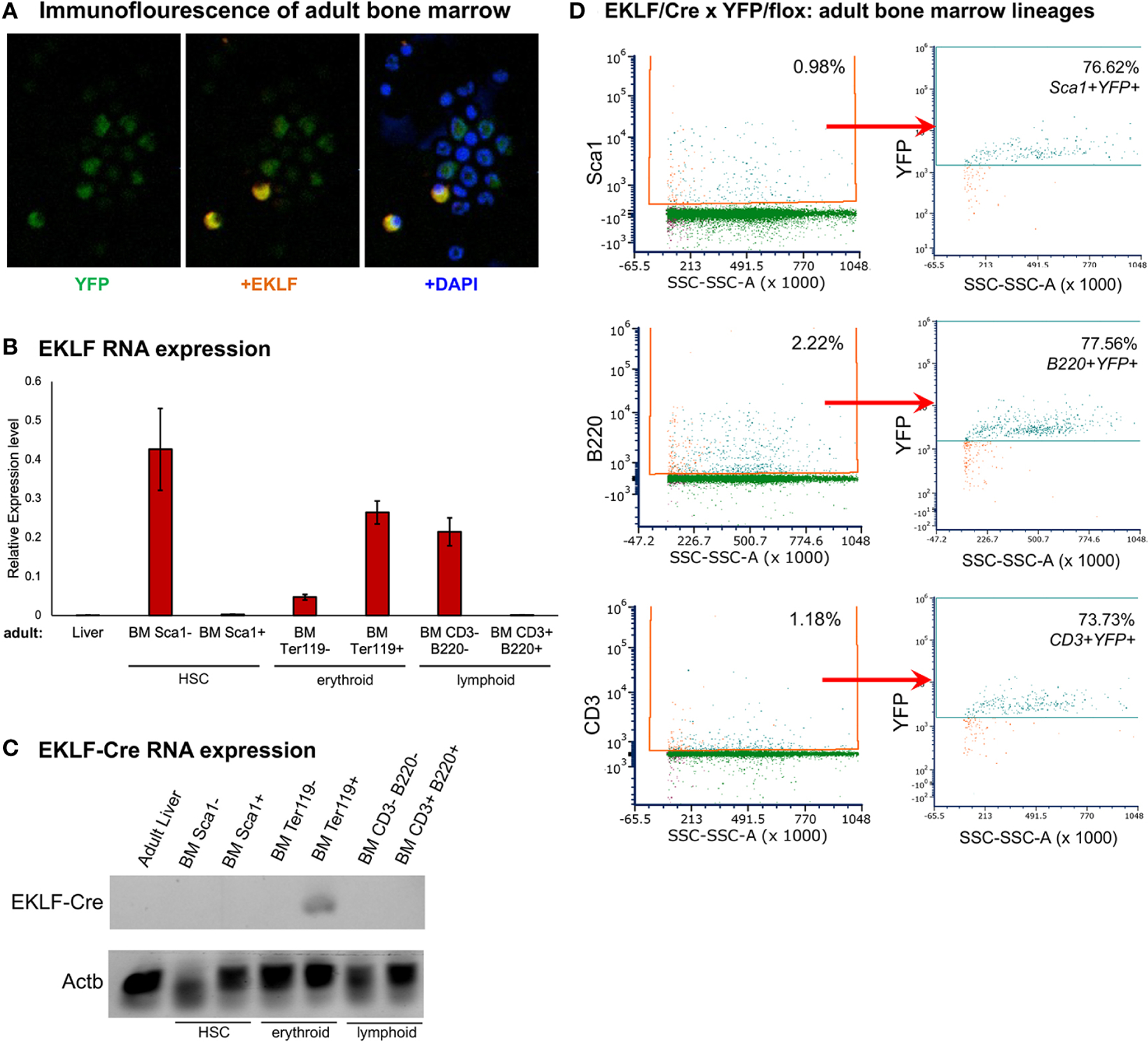
Lineage analysis of adult bone marrow cell populations. (**A**) Immunofluorescence analysis of overlapping expression patterns, showing YFP (EKLF) in *green*, EKLF in *red*, and nuclear stain (dapi) in *blue*. A subset of multiple fields that totaled >450 cells is shown; these show that ~30% BM cells are YFP+, and ~4% are EKLF+. All EKLF+ cells are positive for YFP. (**B**) RT-qPCR analysis of EKLF expression in cells enriched by magnetic beads using cell surface markers for HSCs (Sca1), erythroid (Ter119), or lymphoid (CD3/B220) cells. (+) and (−) populations were analyzed separately, and adult liver cells served as a negative control. (**C**) Semi-quantitative analysis of EKLF-CRE RNA in magnetic bead-enriched cells as in (**B**). Primers used were Cre-F/3A (Figure 1). ß-actin (Actb) was used as a normalization control for all samples. (**D**) FACS analysis of YFP expression overlap with HSCs (Sca1) or lymphoid (CD3 and B220) cells. Percentages of each cell type is shown. Gating parameters for this experiment are shown in Supplementary Figure S3, and purity of the sorted populations was verified by RT-qPCR analysis (Supplementary Figure S4).

The level of adult BM YFP+ cells that are CD71-/Ter119- is not completely accounted for by F4/80+ macrophage cells. We investigated this more carefully, initially by immunofluorescence (IF) where we find that, as expected, not all bone marrow cells are YFP+; surprisingly however, not all YFP+ are EKLF+ (Figure 6A).

The onset of EKLF expression during hematopoiesis begins prior to the megakaryocyte-erythroid progenitor (MEP) stage within the multipotent progenitor (MPP) and retained in the myeloid progenitor (CMP) but not the lymphoid progenitor (CLP) (14, 15). We FACS-sorted adult BM populations by using Sca1 as a cell surface marker for HSCs, B220 for B cells, and CD3 for T cells, and verified by RNA analysis that EKLF expression resides in the Sca1-, CD3-, and B220-populations (Figure 6B). As a positive control, Ter119+ erythroid cells were found to be highly enriched for expression (Figure 6B), consistent with the earlier analyses (Figure 3). In addition and importantly, using primers specific for the EKLF-CRE fusion RNA product, we find expression only within the Ter119+ cell population (Figure 6C). These results verify the expected tissue-specificity of EKLF RNA expression and that of the knock-in fusion variant.

These data suggest that our IF observations follow from a lineage trace that began within the first hematopoietic cell that expresses EKLF. In our knock-in mice, such early expression of EKLF-CRE will stably mark those cells and YFP expression will be retained in any progeny. We tested this idea by FACS sorting, which shows that YFP+ cells are present within a majority (~75%) of Sca1+ HSCs (or MPPs) and progeny that includes B (B220+) and T (CD3+) cell populations (Figure 6D, Supplementary Figure S3). The purity of the cell types was verified by RT-qPCR analysis of the sorted populations (Supplementary Figure S4).

These data fill in the contributions of EKLF-CRE expression to YFP positivity in adult BM marrow cell populations and support prior analyses that showed EKLF expression begins early within the hematopoietic hierarchy, likely within the MPP or even the HSC and thus prior to the CMP/CLP split. Even though EKLF expression is restricted to and expanded within the erythroid lineage, remnants of its early hematopoietic onset remain visible by YFP tracing.

## Discussion

We have taken advantage of EKLF’s restricted localization to generate a novel mouse reagent for directed expression of CRE recombinase. This property, established via a knock-in strategy, provides it with an inherent advantage over other CRE designs that have relied on transgenic approaches to marking cells. The inclusion of a cis-cleaving linker between CRE and the 3’ end of the EKLF coding region circumvents potential complications from haploinsufficiency or from interference with EKLF function.

The highly penetrant yet restricted CRE function in the early developing embryo as well as in the adult demonstrates the versatility of this strain. We have expanded upon prior observations to show that EKLF is expressed within the island F4/80+ macrophage in adult bone marrow. The F4/80+ population is heterogeneous, and it is likely that CRE is expressed only within a subset of these cells, as was the case for EKLF expression in fetal liver F4/80+ cells (20). Our present analyses also support this idea.

Our earlier studies showing EKLF expression early in hematopoiesis utilized Lin-Kit+Sca1+Thy1.1-Flk2- cell surface markers for prospective sorting of MPPs from bone marrow (14). However, as Sca1 expression is also seen in LT-HSCs (41), our present data can be construed to suggest HSCs also express EKLF. There have been other reports that EKLF is expressed at low levels in HSCs (42–44), including those derived from mouse fetal livers (45), where downstream committed cell population numbers are altered in its absence, On the other hand, increased EKLF expression in HSCs is found in cells with activated NF-kB, where EKLF represses c-Mpl expression, leading to reduced HSC quiescence and activation of an erythroid-enriched program emanating from these progenitors (46).

We suggest that this mouse line will be useful for analogous studies as presently described and will also serve to complement other recent recombinase mouse models (e.g., G1BCreER (47)) and EpoR-tdTomato-Cre (48)).

## Supplementary Material

1

## Data Availability

The original contributions presented in the study are included in the article/Supplementary Material. Further inquiries can be directed to the corresponding author.

## Highlights

- Mice with CRE expression under the control of the EKLF promoter have been established.
- These exhibit highly penetrant CRE activity that precisely mimics endogenous EKLF expression patterns in fetal liver and adult erythroid cells as well as in island macrophages.
- These mice have been used to demonstrate that adult bone marrow macrophages retain similar phenotypic properties as their fetal liver counterpart.
