# Supplementary material for: Generation, characterization, and use of EKLF(Klf1)/CRE knock-in mice for cell-restricted analyses
Source: Front Hematol. Author manuscript; Available in PMC 2024 Sep 13. (PMC11393758; doi:10.3389/frhem.2023.1292589)
Supplement: 1 [file NIHMS1969873-supplement-1.pdf]

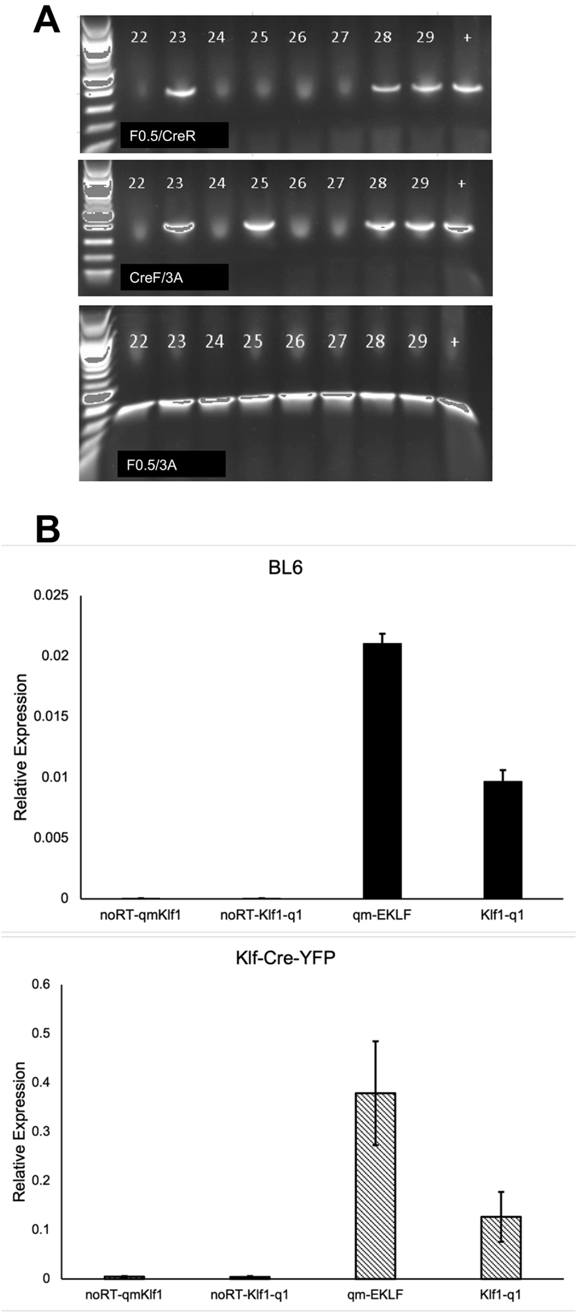

Supplemental Figure S1. Genomic and RNA analyses of newly-established EKLF-CRE lines.

A) Genomic DNA derived from littermate tails were analyzed for correct presence of 5’ (F0.5/CreR; 354 bp) and 3’ (CreF/3A; 377 bp) junctions between endogenous EKLF and the linker/T2A/linker/CRE donor insert (Fig 1). A separate analysis with F0.5/3A yields a 321 bp amplicon if the allele is WT (ie, no knock-in). In this case, lines 23, 28, and 29 have both junction products, hence positive; these are also hets. Molecular weight markers are on the left.

B) RT-qPCR analysis of EKLF expression in bone marrow RNA from a Bl6 mouse (top) compared to that from an EKLF-CRE mouse (bottom). Data is shown from technical triplicates after analyses using two different primer pairs for EKLF RNA detection (qmKlf1 and Klf1-q1, as indicated), relative to that from ß-actin. Note the scale difference in the y-axis.

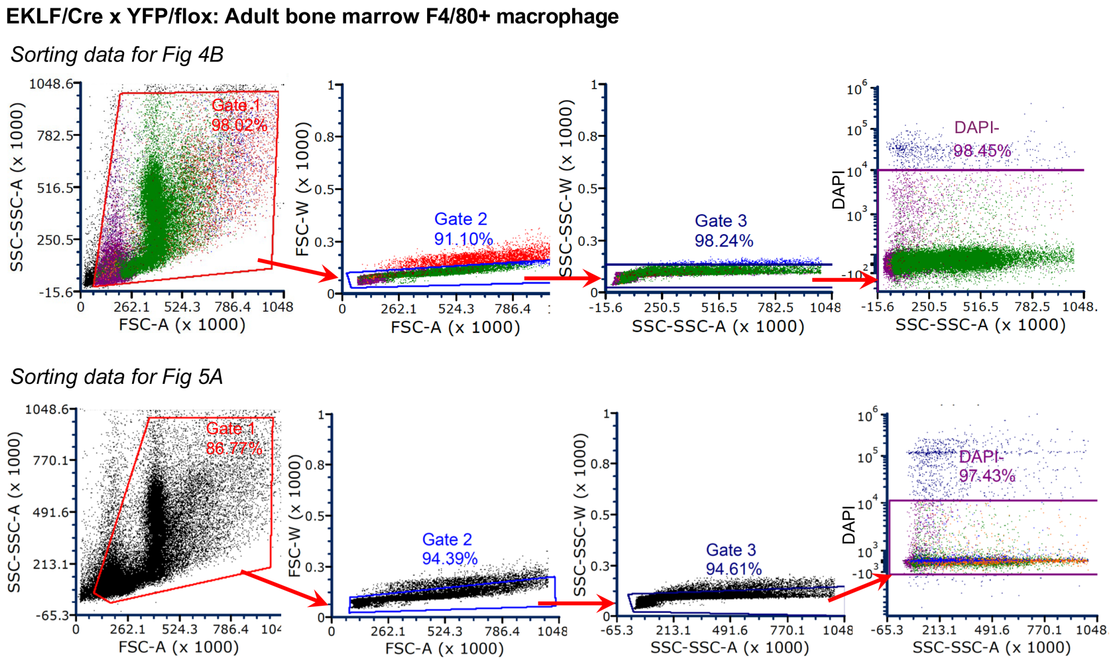

Supplemental Figure S2. Gating strategy for adult bone marrow F4/80+ macrophage. SSC, FSC, and DAPI sorting gates used for YFP and F4/80 analysis in Fig 4B is shown on top, and those used for YFP, F4/80, and Sptb analysis in Fig 5A are shown on bottom.

EKLF/Cre x YFP/flox: adult bone marrow lineages

Sorting data for Fig 6

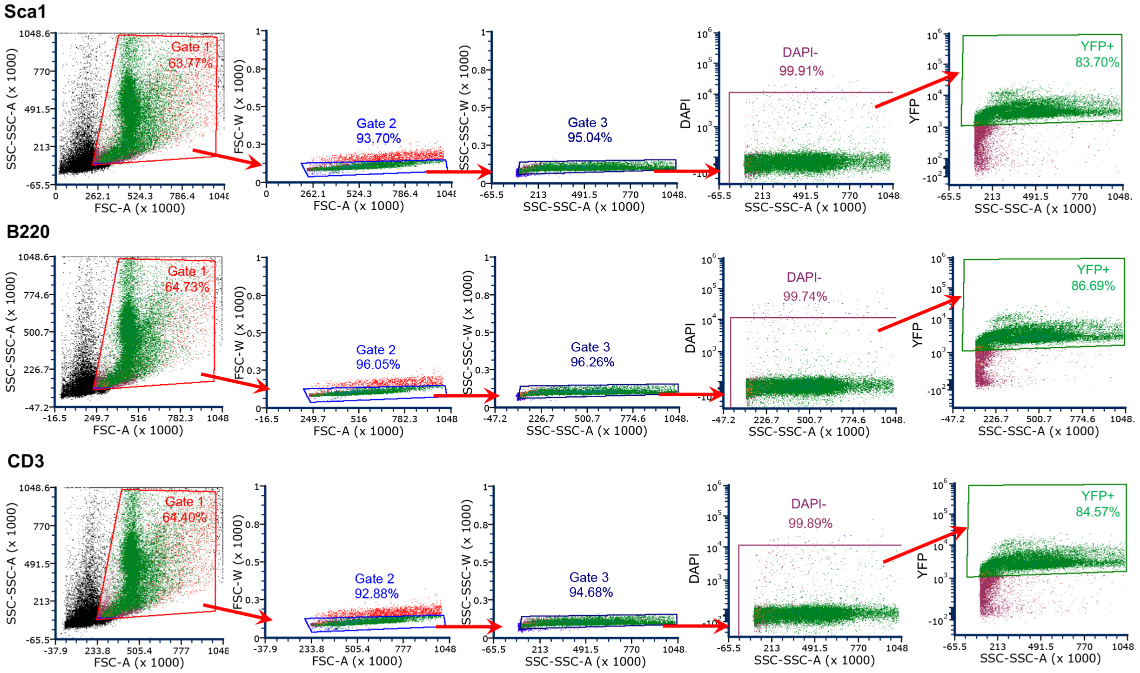

Supplemental Figure S3. Gating strategies for adult bone marrow hematopoietic stem cells and lymphoid cells.

SSC, FSC, and DAPI sorting gates used for YFP isolation and analysis in Fig 6 is shown for Sca1, B220, and CD3.

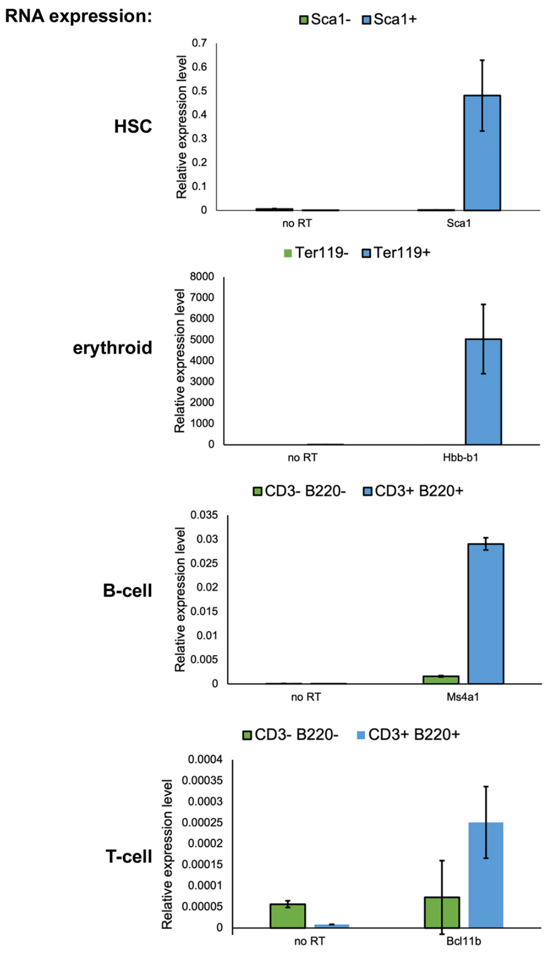

Supplemental Figure S4. RNA analysis of sorted populations.

Results from RT-qPCR analyses of cells from sorts as in Figs 6 and S3 are shown using gene-specific primers for Sca1 (for Sca1+/- HSCs), Hbb-b1 (for Ter119+/- erythroid cells), Ms4a1 (for B220+/- B-cells), and Bcl11b (for CD3+/- T-cells).

Supplemental Table S1

DNA oligos:
F0.5
TCCTGGGACGGCTGTGAC
CreR
CTGCCTGTCCCTGAACATGT
3A
GGCCATGTGTGGGGCTCC
CreF
GAAATCATGCAGGCTGGTGG
YFP-F
GCACGACTTCTTCAAGTCCG
YFP-R
CGGATCTTGAAGTTCACCTTG
mSca1F
AGGAGGCAGCAGTTATTGTGG
mSca1R
CGTTGACCTTAGTACCCAGGA
mMs4a1F
AACCTGCTCCAAAAGTGAACC
mMs4a1R
CCCAGGGTAATATGGAAGAGGC
mBcl11bF
CCCGACCCTGATCTACTCAC
mBcl11bR
GGAGGTGGACTGCTCTTGT
qmActb-F
GGCTGTATTCCCCTCCATCG
qmActb-R
CCAGTTGGTAACAATGCCATGT
qm-EKLF-F
GGACACCCAGGAGGACTTC
qm-EKLF-R
GGGTCCTCCGATTTCAGACTCAC
Klf1-q1-F
ACATCGTCCCTTCTGCTGTG
Klf1-q1-R
TTCATCCCCAGTCCTTGTGC
Hbb-b1-F
CAGCCTCAGTGAGCTCCACTG
Hbb-b1-R
GATCATATTGCCCAGGAGCC

gRNA:
/AltR1/rArUrCrUrUrUrUrGrGrGrArUrArCrGrGrUrCrCrUrGrUrUrUrUrArGrArGrCrU
rArUrGrCrU/AltR2/
[based on ATCTTTTGGGATACGGTCCTTGG]

Donor DNA:
EKLF-linker(GSGG)-T2A(EGRGSLLTCGDVEENPG/P)-linker(GS)-iCRE-EKLF
[iCRE based on pGAG-iCre (ref 27), SV40 NLS is *italicized*]
[gDNA sequence is underlined; PAM site is not present in construct]

AGGTTCGCTCGCTCAGACGAACTGACGCGCCACTACCGGAAGCACACTGGACATCGTCCCTTCTGCTGT
GGCCTCTGCCCACGTGCTTTTTCACGCTCTGACCACTTAGCTCTGCACATGAAGCGTCACCTCGGCTCC
GGCGGCGAGGGCAGAGGCTCCCTGCTGACCTGCGGCGACGTGGAGGAGAACCCCGGCCCCGGATCTATG
GTG*CCCAAGAAGAAGAGGAAAGTC*TCCAACCTGCTGACTGTGCACCAAAACCTGCCTGCCCTCCCTGTG
GATGCCACCTCTGATGAAGTCAGGAAGAACCTGATGGACATGTTCAGGGACAGGCAGGCCTTCTCTGAA
CACACCTGGAAGATGCTCCTGTCTGTGTGCAGATCCTGGGCTGCCTGGTGCAAGCTGAACAACAGGAAA
TGGTTCCCTGCTGAACCTGAGGATGTGAGGGACTACCTCCTGTACCTGCAAGCCAGAGGCCTGGCTGTG
AAGACCATCCAACAGCACCTGGGCCAGCTCAACATGCTGCACAGGAGATCTGGCCTGCCTCGCCCTTCT
GACTCCAATGCTGTGTCCCTGGTGATGAGGAGAATCAGAAAGGAGAATGTGGATGCTGGGGAGAGAGCC
AAGCAGGCCCTGGCCTTTGAACGCACTGACTTTGACCAAGTCAGATCCCTGATGGAGAACTCTGACAGA
TGCCAGGACATCAGGAACCTGGCCTTCCTGGGCATTGCCTACAACACCCTGCTGCGCATTGCCGAAATT
GCCAGAATCAGAGTGAAGGACATCTCCCGCACCGATGGTGGGAGAATGCTGATCCACATTGGCAGGACC
AAGACCCTGGTGTCCACAGCTGGTGTGGAGAAGGCCCTGTCCCTGGGGGTTACCAAGCTGGTGGAGAGA
TGGATCTCTGTGTCTGGTGTGGCTGATGACCCCAACAACTACCTGTTCTGCCGGGTCAGAAAGAATGGT
GTGGCTGCCCCTTCTGCCACCTCCCAACTGTCCACCCGGGCCCTGGAAGGGATCTTTGAGGCCACCCAC
CGCCTGATCTATGGTGCCAAGGATGACTCTGGGCAGAGATACCTGGCCTGGTCTGGCCACTCTGCCAGA
GTGGGTGCTGCCAGGGACATGGCCAGGGCTGGTGTGTCCATCCCTGAAATCATGCAGGCTGGTGGCTGG
ACCAATGTGAACATTGTGATGAACTACATCAGAAACCTGGACTCTGAGACTGGGGCCATGGTGAGGCTG
CTCGAGGATGGGGACTGACACAAATACCACTGAGATCTTTTTCCCTCTGCCAAAAATTATGGGGACATC
ATGAAGCCCCTTGAGCATCTGACTTCTGGCTAATAAAGGAAATTTATTTTCATTGCAATAGTGTGTTGG
AATTTTTTGTGTCTCTCACTCGGAACCGTATCCCAAAAGATGAGCCATTATATAGTCCTACCCAGATCA
AAAACTGACCAGAAGACCATACAAAGGAGCCTTCAGGACAAACCTCACATGTCCTCAGGGAGCCCCACA
CATGGC
